# The Bounds Of Education In The Human Brain Connectome

**DOI:** 10.1038/srep12812

**Published:** 2015-08-06

**Authors:** P. Marques, J. M. Soares, R. Magalhães, N. C. Santos, N. Sousa

**Affiliations:** 1 Life and Health Sciences Research Institute (ICVS), School of Health Sciences, University of Minho, Campus Gualtar, 4710-057 Braga, Portugal; 2 ICVS/3B’s - PT Government Associate Laboratory, Braga/Guimarães, Portugal; 3 Clinical Academic Center – Braga, Braga, Portugal

## Abstract

Inter-individual heterogeneity is evident in aging; education level is known to contribute for this heterogeneity. Using a cross-sectional study design and network inference applied to resting-state fMRI data, we show that aging was associated with decreased functional connectivity in a large cortical network. On the other hand, education level, as measured by years of formal education, produced an opposite effect on the long-term. These results demonstrate the increased brain efficiency in individuals with higher education level that may mitigate the impact of age on brain functional connectivity.

Normal aging is characterized by inter-individual heterogeneity with some individuals showing a marked (cognitive) decline, while others evidence relatively preserved cognition^1^,^2^. One of the factors thought to contribute for this heterogeneity is education level, a factor associated to “cognitive reserve” (CR). CR can be perceived as the brain’s capacity to optimize/maximize performance^3^. More so, previous studies have shown that older individuals with higher education levels evidence larger brains and lower levels of brain activity during task performance, thus reflecting more efficient brain networks^4^.

One of the recent trends in the neuroimaging field is the use of graph theory models to study the human brain connectome as a network, modeling each brain region as a node and the connections between brain regions as the edges of a graph^5^. Resting-state functional magnetic resonance imaging (rs-fMRI) is one of the most widely used neuroimaging techniques to study the brain’s functional connectivity (FC) and has been used in combination with graph theory to study the human functional connectome in health^6^ and disease^7^. Concerning aging, it has been recently shown that it has a negative impact on the FC of several brain networks such as the default mode network (DMN) and the salience network as well as in internetwork connectivity, with these changes being associated with cognitive decline^8^. At the connectome level, there is evidence that long-range connections are negatively impacted by aging, while short-range connection may display higher FC with aging^9^. Comparative studies between older and younger individuals, using graph theory, have shown that aging is associated with decreased network efficiency in frontal and temporal regions and a rearrangement in the modular organization of the brain^10^,^11^.

Yet, to the best of our knowledge, no study has yet characterized the effect of education level, one of the major proxies of CR, in the brain’s functional network in aging. We performed rs-fMRI on 97 healthy older subjects (without manifestation or diagnosed brain pathology), modeling whole FC patterns and investigating: (i) the cross-sectional effects of healthy aging in the human functional connectome, and (ii) the effects of education level in the human connectome as well as possible interactions with the aging effects.

## Results

### Demographic Characterization

Table 1 presents the demographic characteristics of the sample under study, separated by sex and also presents the p-value of two-sample T-tests performed between males and females. Males and females were matched for age, with males presenting significantly higher number of years of formal education (p < 0.001) and MMSE scores (p < 0.001), when compared to females. Additionally, years of formal education were positively correlated with MMSE scores (r = 0.36; p < 0.001) and negatively correlated with age (r = –0.23; p = 0.022).

### Associations between age and FC networks

**Figure 1:**
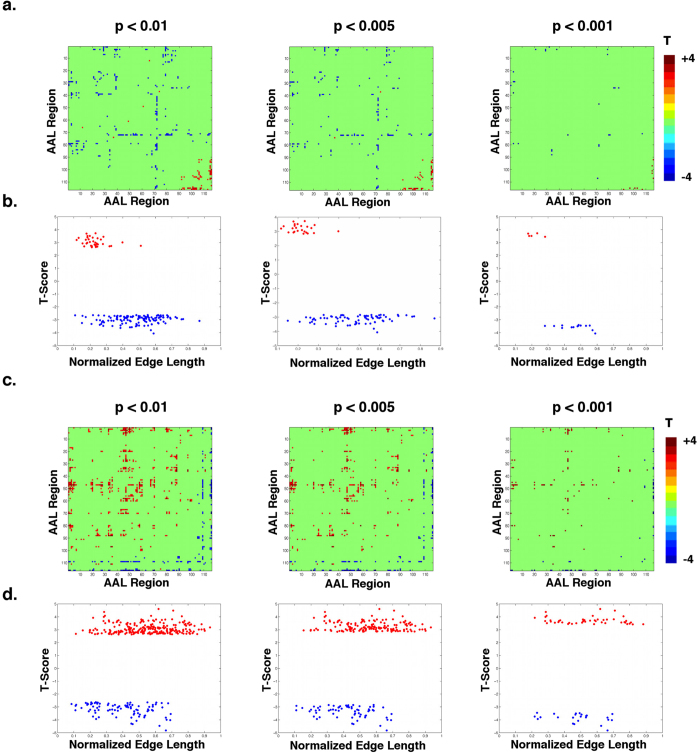
Functional connectivity matrices and edge length distribution of the edges surviving the primary thresholding procedure. Both positive and negative associations with age survive the three (p < 0.01, p < 0.005, p < 0.001) primary thresholds of the NBS analysis (**a**). Positive associations with age occur mainly in short-range connections while negative associations can be found in medium to long-range edges (**b**). Similarly, positive and negative associations with years of formal education survive the application of the primary thresholds (**c**) and the supra-threshold edges span a large range of lengths (**d**).

Several edges revealed associations with aging that survived the three primary thresholds (p < 0.01, p < 0.005, p < 0.001) used in the Network Based-Statistic (NBS) analysis (Fig. 1a). Both positive and negative associations survived the thresholding procedure. From these edges, the ones exhibiting positive associations with age were mainly short-range connections while negative associations were found for a wide range of normalized edge lengths (Fig. 1b).

**Figure 2:**
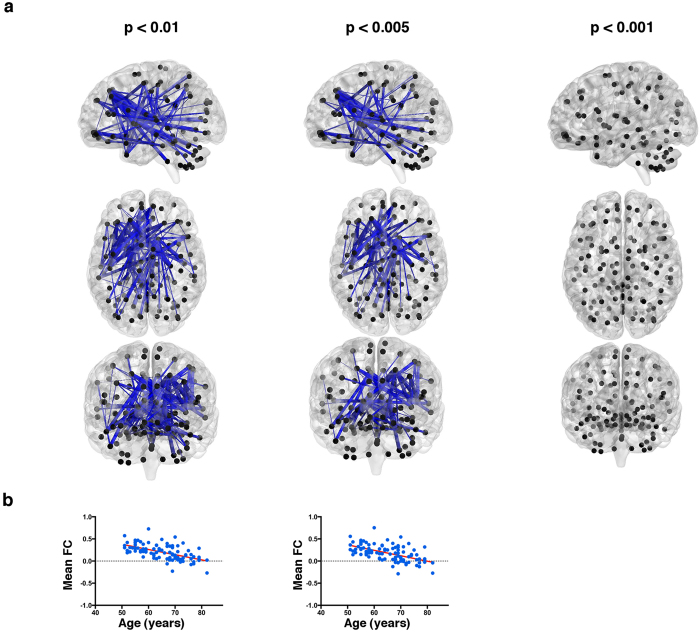
Networks showing significant associations between functional connectivity (FC) and age. A large sub-network was found to display a negative association between FC and aging using primary thresholds of p < 0.01 and p < 0.005 but not with a primary threshold of p < 0.001 (**a**). Younger subjects presented moderate positive mean FC values in this network while older subjects presented mean FC values close to zero along the network (**b**). In the upper panel (**a**), black dots represent AAL brain regions and blue edges denote edges with negative associations with age. Bottom panel (**b**) presents scatter plots of the mean FC of the statistically significant networks in function of age.

Regarding NBS analysis, age presented a linear negative relationship with FC in a large and extent sub-network (Fig. 2a) when using the most liberal primary threshold (i.e. p < 0.01). This effect was still evident for a more restricted primary threshold (i.e. p < 0.005), but was not significant for the most restricted threshold (p < 0.001). The bilateral caudate, left frontal superior, left Heshl and left parahipoccampal were the brain regions through which most of the connections passed (the complete list of node degrees can be consulted in Supplementary Table S1 and Supplementary Table S2). Although both positive and negative associations between edges and age survived the primary threshold, the edges with positive associations did not survive the NBS procedure (network level inference), meaning that these positive associations do not form a significant network. Furthermore, interpreting the scatter plots between mean FC in the significant networks and age (Fig. 2b), younger individuals presented moderate positive mean FC Z-scores while older individuals present mean FC Z-scores of approximately zero.

### Associations between years of formal education and FC networks

Regarding the effects of years of formal education, several edges survived the application of the three primary thresholds (Fig. 1c). In Fig. 1d it is noted that these edges span a broad range of edge lengths, both in positive and negative associations.

**Figure 3:**
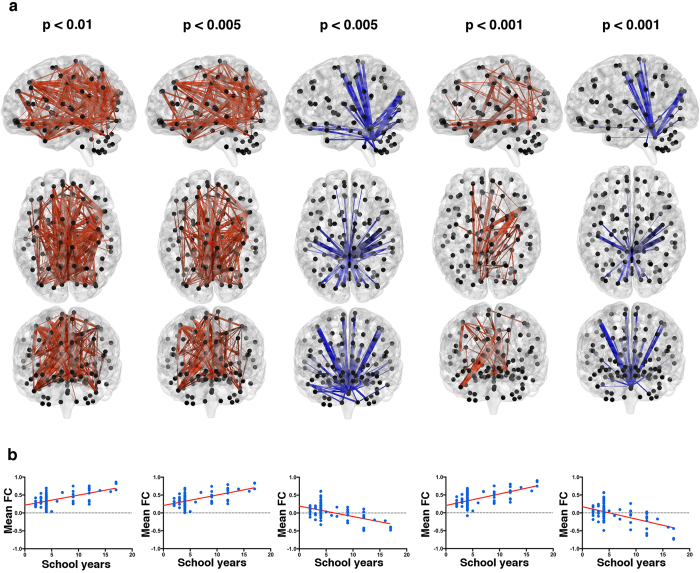
Networks showing significant associations between functional connectivity (FC) and years of education. A large sub-network showed a positive association between FC and years of formal education using primary thresholds of p < 0.01, p < 0.005 and p < 0.001. A focal sub-network displayed a negative association with years of education using a primary threshold of p < 0.005 and p < 0.001 (**a**). In the network that evidenced a positive association with FC, less educated subjects presented moderately low positive FC values while more educated subjects presented moderately high positive FC values (**b**). In the network with a negative association with years of formal education, mean FC ranges from low positive FC values to moderate negative mean FC values from individual with lower years of formal education to individuals with higher number of years of formal education (**b**). In the top panel (**a**) black dots represent AAL brain regions and blue and red edges denote edges with negative and positive associations with years of education, respectively. Bottom panel (**b**) presents scatter plots of the mean FC of the statistically significant networks in function of years of education.

The NBS results for the associations between FC networks and years of formal education are shown in Fig. 3a. Results revealed that a large and distributed network showed a positive association between FC and years of formal education when using the most liberal primary threshold (p < 0.01). When using a stricter primary threshold (p < 0.005), a large sub-part of the previous network still evidenced a positive association with education level. Concerning strong focal effects of education level on FC (p < 0.001), a sub-network still revealed a positive association with education level. Regarding the contribution of each region for this network, the left lingual, left frontal superior, right precentral, left cuneus and the middle right temporal pole middle were some of the main contributors (the complete list of node degrees can be consulted in Supplementary Table S3, Supplementary Table S4 and Supplementary Table S5).

Furthermore, a focal network that was not significant with the most liberal primary threshold used (p < 0.01), evidenced a negative association with years of formal education when using the stricter primary thresholds (p < 0.005 and p < 0.001). This was a highly bilateral network with several connection from/to the Vermis 10 and Vermis 12 (full list of node degrees can be found in Supplementary Table S6 and Supplementary Table S7).

Figure 3b reveals that in the network with positive associations with years of education, the mean FC seems to increase from low positive values to high positive values, while in the network evidencing a negative associations with years of formal education, the mean FC seems to decrease from low positive values to moderate negative values.

No interactions were found between years of formal education and age in FC networks.

## Discussion

The present work aimed at exploring the cross-sectional effects of healthy aging and education level in the brain’s FC. In order to achieve this, a large cohort of healthy older individuals was screened so to investigate FC patterns with network-based inference applied to rs-fMRI data.

Concerning the aging effect, age was associated with a decrease in FC. This effect was rather global as no sparse or focal sub-network was found to present an increase in FC with increasing age. A recent study analyzed a similar population in terms of mean age and age range finding both increases and decreases in FC^9^. With a similar threshold (i.e. primary threshold of p < 0.001), here only a few connections were found to display such pattern; however, when making inferences at the network level, these did not form a significant sub-network. These differences might arise either from the fact that, in the referred study, correction for multiple comparisons was not performed at the network level or, most likely, these connections that display positive associations with age do not form a network at all. Additionally, the magnetic field strength of the MRI scanner and the resolution of acquisition were different from those used in the present study, which could potentially justify part of these discrepancies given the greater signal-to-noise ratio of the 3T MRI scanners^12^. Furthermore, here the results are compatible with the cognitive decline commonly associated with the aging pattern^13^,^14^,^15^. Reduced FC might reflect an overall greater disconnection between several brain regions with repercussions in brain function, such as slower processing speed^16^. Interestingly, findings here are also in line with the posterior-to-anterior shift with aging (PASA) theory^17^, as a greater disconnection with increasing age in the anterior parts of the brain was found. The PASA pattern is characterized by greater frontal activity in older individuals, compared to younger individuals in order to perform the same task, which is likely to reflect a compensatory mechanism. Additionally, reductions in deactivation in midline posterior regions and increased deactivation in midline anterior regions have also been observed^18^. Davis *et al.*^18^ hypothesize that this deactivation shift could reflect an increased demand in older individuals to reallocate resources from the Default Mode Network (DMN) for task performance. Our findings of reduced FC in anterior connections in resting conditions could justify an increased effort in task conditions in order to compensate for a greater FC disconnection.

Regarding the effects of education level (in this work assessed as years of formal education), we demonstrated that it has has a strong positive effect on the FC of a large network. Unlike the aging pattern, this network involves regions and edges located in all lobes and both hemispheres with the exception of the cerebellum. These results are in line with the CR theory^3^, in which education level is critical, since a greater FC in this large network is likely to reflect a more finely tuned brain network, easing communication and integration of information, and thus requiring less effort to perform cognitive tasks for those with higher education level^19^. Importantly, no interaction between years of education and age was found, providing further evidence to the inexistence of a mediating effect of education level in rates of decline^20^. Rs-fMRI studies have shown education to be a modulator of FC within the DMN in Alzheimer’s disease patients, particularly with the posterior cingulate cortex; however they have not indicated significant associations between FC and education in healthy controls^21^. Collectively, it could thus be expected that the posterior cingulate cortex would not be involved in the networks that were here found to display positive associations with years of formal education, which was not the case. However, on this, several aspects must be noted. The sample of healthy controls used in the study from Bozzali and colleagues^21^ was much smaller than the current one and, possibly, their sample size was not enough to achieve statistical significance. Additionally, herein connectivity at the whole brain network level was explored while Bozzali and colleagues^21^ explored connectivity, specifically with the DMN.

Intriguingly, here it was found a particular network involving connections (mostly bilateral) linking parts of the cerebellum vermis to cerebral regions in the occipital and temporal lobes, postcentral and precentral regions, rectus and cingulum middle regions, which displayed a negative association with years of education. The classical view that the cerebellum is solely implicated to motor control has long been discarded^22^; with evidence from several studies involving healthy individuals and patients with cerebellar lesions, indicating the involvement of the cerebellum in cognition and emotion^23^,^24^. In the context of these evidences, the finding of associations between FC and education level are not particularly unexpected; here, the intriguing finding concerns the fact that these are negative associations. However, when looking at the mean FC of this network, it can be observed that in absolute values it is actually positively associated with years of education (meaning that increased education level is associated with increased anti-correlations ,in this network, rather than the loss of FC). Given that there is evidence for the differential involvement of the cortex and the cerebellum and for education to modulate this functional balance^25^, it is hypothesized that this negative association does not reflect a negative impact but rather a more effective involvement of the cerebrum and cerebellum through this network.

A major limitation of the present study is its cross-sectional design. A longitudinal approach would enable the within-subject analysis of the effect of aging, thus reducing inter-individual variability. This is particularly critical regarding the possible mediating effects of education level in the associations between FC and aging.

In summary, the present work brings further evidence regarding the deleterious effect of aging through the loss of FC in a large sub-network of the brain. Importantly, we show for the first time that a higher educational level is associated with increased FC in a large cortical brain network, possibly reflecting greater neural efficiency, which may contribute to ‘alleviate’ the deleterious impact of increasing age in brain function.

## Methods

### Participants

The present study was performed with a sample comprising 97 participants (49 males, 48 females; mean age 64.94 ± 8.20, minimum age 51, maximum age 82; mean years of education 5.37 ± 3.83, minimum 0, maximum 17 years of education), after inclusion/exclusion criteria were met. All participants underwent an extensive battery of neuropsychological and neurocognitive tests in order to rule out the presence of Mild Cognitive Impairment and dementia. In the recruitment phase, exclusion criteria including inability to understand informed consent, participant choice to withdraw from the study, incapacity and/or inability to attend the clinical and neuropsychological assessment session(s), dementia and/or diagnosed neuropsychiatric and/or neurodegenerative disorder (including via verification from medical records) were verified by a clinician. Additionally, individuals presenting MMSE scores below the adjusted thresholds for cognitive impairment were not included in the present study. Regarding the MMSE scores, recommendations state that the corresponding threshold should be adjusted depending on factors such as age and/or education. The following adjusted thresholds for cognitive impairment were calculated and applied: MMSE score <17 if individual with ≤4 years of formal school education and/or ≥72 years of age, and MMSE score <23 otherwise [follows the MMSE validation study for the Portuguese population^26^].

This sample is part of a cohort of subjects that was recruited for the SWITCHBOX Consortium project (www.switchbox-online.eu/) and is representative of the Portuguese older population in terms of age, education and sex^27^. The study goals and tests were explained to all participants, which gave informed written consent. The study was conducted in accordance with the principles expressed in the Declaration of Helsinki and was approved by the Ethics Committee of Hospital de Braga (Portugal).

### Image Acquisition

Imaging was performed at Hospital de Braga on a clinical approved 1.5 T Siemens Magnetom Avanto MRI scanner (Siemens, Erlangen, Germany). A structural T1 scan (176 sagittal slices, TR/TE = 2730/ 3.48 ms, FA = 7°, slice thickness = 1 mm, slice gap = 0 mm, voxel size = 1 × 1 mm2, FoV = 256 mm) and a functional blood oxygen level dependent (BOLD) sensitive echo-planar imaging (EPI) scan (30 axial slices, TR/TE = 2000/30 ms, FA = 90°, slice thickness = 3.5, slice gap = 0.48 mm, voxel size = 3.5 × 3.5 mm2 FoV = 1344 mm and 180 volumes) were collected. During the resting state scan, the subjects were instructed to remain still, awake, with their eyes closed, as motionless as possible and to think of nothing in particular. None of the participants fell asleep during the acquisition.

### Image Preprocessing

Image pre-processing was performed with tools provided with the FMRIB Software Library (FSL v5.07; http://fsl.fmrib.ox.ac.uk/fsl/). Preprocessing steps included: the removal of the first five volumes of the acquisition; slice-timing correction; motion correction; normalization to MNI standard space; regression of motion parameters, mean WM and CSF signals; band-pass temporal filtering (0.01–0.08 Hz). For each subject, mean time-series for 116 cortical, subcortical and cerebellar regions from the Anatomical Automatic Labeling (AAL) atlas^28^ were then extracted by averaging all voxels composing each region for each time point. Pearson correlation coefficients were then calculated between the time-series of each pair of regions, resulting in one adjacency matrix per subject. These matrices were then transformed to Z-score matrices by the application of Fisher’s r-to-Z transform to the correlation coefficients.

### Network Analysis

Each adjacency matrix represents the entire functional brain network of the corresponding subject, with the vertices of the network being the AAL regions and the edges the correlations between regions’ time-series. Herein we aimed at exploring the cross-sectional effects of age and years of education, on brain’s functional network. In order to achieve this we built a General Linear Model (GLM) including the linear and quadratic effect of age and the linear effect of years of education as regressors. We decided to include both linear and quadratic effect of age given the relatively large range of age (brain regions are known to follow nonlinear aging effects^29^). Additionally, we also included MMSE scores in the model in order to check/control for cognition associations. Sex was included as covariate in the model in order to control for its’ effect. Additionally, we built a second model in which we added the age*years of education interaction terms in order to investigate if cognitive reserve could be a modulator of the aging effect in FC. Correlations between every pair of regressors were performed in order to check for collinearity issues and none were collinear.

Statistical analysis was performed at the network level through the use of Network-Based Statistics (NBS)^30^. This approach increases statistical power by finding sub-networks that, as whole, display a similar pattern regarding a contrast of interest and enabling inference while controlling for family-wise error (FWE) rate. This procedure is similar to cluster-based inference of the more typical voxel-wise fMRI analysis. Specifically, initially, the model was fitted to every edge of the whole brain network. Then, edges with a p-value less than a primary uncorrected threshold (e.g. p < 0.005) obtained for a particular effect (e.g. age effect) were filtered in. Based on these supra-threshold edges, components or subnetworks of interconnected edges were identified and their size was calculated. Then 5000 permutations were performed in order to build a null distribution for the largest subnetwork size. Significance of each subnetwork was calculated as the percentage of permutations that originated subnetworks with greater size than the originally identified subnetwork.

One of the difficulties in such analysis is the definition of the primary threshold. The authors of the NBS procedures recommend the use of a range of thresholds since effects present at liberal thresholds (e.g. p < 0.01) are likely to be subtle, yet topologically extended, effects while effects captured using more strict thresholds (e.g. p < 0.001) reflect more focal and strong effects. In order to capture both kinds of effects, three primary thresholds (p < 0.01, p < 0.005 and p < 0.001) were used in the present work. Networks were considered significant at a corrected level of p < 0.05 FWE corrected. BrainNet viewer (http://www.nitrc.org/projects/bnv/) was used for visualization purposes.

## Additional Information

**How to cite this article**: Marques, P. *et al.* The Bounds Of Education In The Human Brain Connectome. *Sci. Rep.* **5**, 12812; doi: 10.1038/srep12812 (2015).

## Supplementary Material

Supplementary Information

## Figures and Tables

**Table 1:** Demographic and cognitive characteristics of the participants.

	Total Sample	Male	Females	p-value
	Mean ± SD	Mean ± SD	Mean ± SD	
N	97	49	48	
Age	64.94 ± 8.20	63.73 ± 6.69	66.16 ± 7.90	0.145
Education (years)	5.37 ± 3.83	8.38 ± 4.30	3.91 ± 2.72	<0.001
MMSE	26.54 ± 3.57	27.80 ± 2.26	24.71 ± 4.17	<0.001
