# Supplementary material for: The Bounds Of Education In The Human Brain Connectome
Source: Sci Rep. 2015 Aug 6;5:12812. doi: 10.1038/srep12812 (PMC4526851; doi:10.1038/srep12812)
Supplement: Supplementary Information [file srep12812-s1.pdf]

The Bounds Of Education In The Human Brain Connectome

Authors: P Marques[123], JM Soares[123], R Magalhães[123], NC Santos[123], N Sousa[123*]

[1]Life and Health Sciences Research Institute (ICVS), School of Health Sciences, University of Minho, Campus Gualtar, 4710-057 Braga, Portugal.

[2]ICVS/3B's - PT Government Associate Laboratory, Braga/Guimarães, Portugal.

[3]Clinical Academic Center – Braga, Braga, Portugal.

*Corresponding author: Nuno Sousa, Life and Health Sciences Research Institute (ICVS), School of Health Sciences, University of Minho, Campus Gualtar, 4710-057 Braga, Portugal. Email: njcsousa@ecsaude.uminho.pt. Phone: +351 253 604 806.

Supplementary Table S1. Degree of the regions comprising the network that evidenced a negative association with age, using a primary threshhold of p < 0.01.

MNI COORDINATES				
X	Y	Z	Degree	AAL Region
14.84	12.07	9.42	28	Caudate_R
-18.45	34.81	42.2	12	Frontal_Sup_L
-41.99	-18.88	9.98	12	Heschl_L
-21.17	-15.95	-20.7	11	ParaHippocampal_L
-11.46	11	9.24	11	Caudate_L
-33.43	32.73	35.46	8	Frontal_Mid_L
48.25	14.75	-16.86	7	Temporal_Pole_Sup_R
8.16	51.67	-7.13	6	Frontal_Med_Orb_R
-35.13	6.65	3.44	6	Insula_L
8.02	-8.83	39.79	6	Cingulum_Mid_R
29.23	-19.78	-10.33	6	Hippocampus_R
-10.85	-17.56	7.98	6	Thalamus_L
21.9	31.12	43.82	5	Frontal_Sup_R
8.46	37.01	15.84	5	Cingulum_Ant_R
9.98	-56.05	43.77	5	Precuneus_R
-53.16	-20.68	7.13	5	Temporal_Sup_L
-38.65	-5.68	50.94	4	Precentral_L
37.59	33.06	34.04	4	Frontal_Mid_R
-5.17	54.06	-7.4	4	Frontal_Med_Orb_L
41.37	-8.21	52.09	3	Precentral_R
-30.65	50.43	-9.62	3	Frontal_Mid_Orb_L
-45.58	29.91	13.99	3	Frontal_Inf_Tri_L
-8.06	15.05	-11.46	3	Olfactory_L
-4.8	49.17	30.89	3	Frontal_Sup_Medial_L
9.1	50.84	30.22	3	Frontal_Sup_Medial_R
-5.08	37.07	-18.14	3	Rectus_L
-5.48	-14.92	41.57	3	Cingulum_Mid_L
25.38	-15.15	-20.47	3	ParaHippocampal_R
27.32	0.64	-17.5	3	Amygdala_R
45.86	-17.15	10.41	3	Heschl_R
-55.52	-33.8	-2.2	3	Temporal_Mid_L
-36.32	14.59	-34.08	3	Temporal_Pole_Mid_L
44.22	14.55	-32.23	3	Temporal_Pole_Mid_R
17.2	-42.86	-18.15	3	Cerebelum_4_5_R
10.43	15.91	-11.26	2	Olfactory_R
-32.39	-80.73	16.11	2	Occipital_Mid_L
-36.36	-78.29	-7.84	2	Occipital_Inf_L
-31.16	-40.3	-20.23	2	Fusiform_L
-42.46	-22.63	48.92	2	Postcentral_L
57.61	-31.5	34.48	2	SupraMarginal_R
-44.14	-60.82	35.59	2	Angular_L
-7.24	-56.07	48.01	2	Precuneus_L
58.15	-21.78	6.8	2	Temporal_Sup_R
-49.77	-28.05	-23.17	2	Temporal_Inf_L
-15	-43.49	-16.93	2	Cerebelum_4_5_L
1.15	-64.43	-34.08	2	Vermis_8
-48.43	12.73	19.02	1	Frontal_Inf_Oper_L

50.2	14.98	21.41	1	Frontal_Inf_Oper_R
-35.98	30.71	-12.11	1	Frontal_Inf_Orb_L
8.35	35.64	-18.04	1	Rectus_R
39.02	6.25	2.08	1	Insula_R
-4.04	35.4	13.95	1	Cingulum_Ant_L
-4.85	-42.92	24.67	1	Cingulum_Post_L
-23.27	-0.67	-17.14	1	Amygdala_L
-7.14	-78.67	6.44	1	Calcarine_L
15.99	-73.15	9.4	1	Calcarine_R
-5.93	-80.13	27.22	1	Cuneus_L
-14.62	-67.56	-4.63	1	Lingual_L
16.29	-66.93	-3.87	1	Lingual_R
38.16	-81.99	-7.61	1	Occipital_Inf_R
33.97	-39.1	-20.18	1	Fusiform_R
-23.45	-59.56	58.96	1	Parietal_Sup_L
26.11	-59.18	62.06	1	Parietal_Sup_R
-55.79	-33.64	30.45	1	SupraMarginal_L
27.78	4.91	2.46	1	Putamen_R
-17.75	-0.03	0.21	1	Pallidum_L
-39.88	15.14	-20.18	1	Temporal_Pole_Sup_L
37.46	-67.14	-29.55	1	Cerebelum_Crus1_R
-23.24	-59.1	-22.13	1	Cerebelum_6_L
24.69	-58.32	-23.65	1	Cerebelum_6_R
-32.36	-59.82	-45.45	1	Cerebelum_7b_L
-22.61	-33.8	-41.76	1	Cerebelum_10_L
1.15	-71.93	-25.14	1	Vermis_7
0.86	-54.87	-34.9	1	Vermis_9
-16.56	47.32	-13.31	0	Frontal_Sup_Orb_L
18.49	48.1	-14.02	0	Frontal_Sup_Orb_R
33.18	52.59	-10.73	0	Frontal_Mid_Orb_R
50.33	30.16	14.17	0	Frontal_Inf_Tri_R
41.22	32.23	-11.91	0	Frontal_Inf_Orb_R
-47.16	-8.48	13.95	0	Rolandic_Oper_L
52.65	-6.25	14.63	0	Rolandic_Oper_R
-5.32	4.85	61.38	0	Supp_Motor_Area_L
8.62	0.17	61.85	0	Supp_Motor_Area_R
7.44	-41.81	21.87	0	Cingulum_Post_R
-25.03	-20.74	-10.13	0	Hippocampus_L
13.51	-79.36	28.23	0	Cuneus_R
-16.54	-84.26	28.17	0	Occipital_Sup_L
24.29	-80.85	30.59	0	Occipital_Sup_R
37.39	-79.7	19.42	0	Occipital_Mid_R
41.43	-25.49	52.55	0	Postcentral_R
-42.8	-45.82	46.74	0	Parietal_Inf_L
46.46	-46.29	49.54	0	Parietal_Inf_R
45.51	-59.98	38.63	0	Angular_R
-7.63	-25.36	70.07	0	Paracentral_Lobule_L
7.48	-31.59	68.09	0	Paracentral_Lobule_R
-23.91	3.86	2.4	0	Putamen_L
21.2	0.18	0.23	0	Pallidum_R
13	-17.55	8.09	0	Thalamus_R
57.47	-37.23	-1.47	0	Temporal_Mid_R
53.69	-31.07	-22.32	0	Temporal_Inf_R

-36.07	-66.72	-28.93	0	Cerebelum_Crus1_L
-28.64	-73.26	-38.2	0	Cerebelum_Crus2_L
32.06	-69.02	-39.95	0	Cerebelum_Crus2_R
-8.8	-37.22	-18.58	0	Cerebelum_3_L
12.32	-34.47	-19.39	0	Cerebelum_3_R
33.14	-63.18	-48.46	0	Cerebelum_7b_R
-25.75	-54.52	-47.68	0	Cerebelum_8_L
25.06	-56.34	-49.47	0	Cerebelum_8_R
-10.95	-48.95	-45.9	0	Cerebelum_9_L
9.46	-49.5	-46.33	0	Cerebelum_9_R
25.99	-33.84	-41.35	0	Cerebelum_10_R
0.76	-38.79	-20.05	0	Vermis_1_2
1.38	-39.93	-11.4	0	Vermis_3
1.22	-52.36	-6.11	0	Vermis_4_5
1.14	-67.06	-15.12	0	Vermis_6
0.36	-45.8	-31.68	0	Vermis_10

Supplementary Table S2. Degree of the regions comprising the network that evidenced a negative association with age, using a primary threshhold of p < 0.005.

MNI COORDINATES				
X	Y	Z	Degree	AAL Region
14.84	12.07	9.42	21	Caudate_R
-41.99	-18.88	9.98	9	Heschl_L
-18.45	34.81	42.2	8	Frontal_Sup_L
-21.17	-15.95	-20.7	8	ParaHippocampal_L
-11.46	11	9.24	7	Caudate_L
-35.13	6.65	3.44	6	Insula_L
21.9	31.12	43.82	4	Frontal_Sup_R
-33.43	32.73	35.46	4	Frontal_Mid_L
8.16	51.67	-7.13	4	Frontal_Med_Orb_R
-53.16	-20.68	7.13	4	Temporal_Sup_L
-38.65	-5.68	50.94	3	Precentral_L
-45.58	29.91	13.99	3	Frontal_Inf_Tri_L
-8.06	15.05	-11.46	3	Olfactory_L
-5.17	54.06	-7.4	3	Frontal_Med_Orb_L
8.02	-8.83	39.79	3	Cingulum_Mid_R
9.1	50.84	30.22	2	Frontal_Sup_Medial_R
-5.08	37.07	-18.14	2	Rectus_L
8.46	37.01	15.84	2	Cingulum_Ant_R
-5.48	-14.92	41.57	2	Cingulum_Mid_L
29.23	-19.78	-10.33	2	Hippocampus_R
25.38	-15.15	-20.47	2	ParaHippocampal_R
-32.39	-80.73	16.11	2	Occipital_Mid_L
-36.36	-78.29	-7.84	2	Occipital_Inf_L
-42.46	-22.63	48.92	2	Postcentral_L
9.98	-56.05	43.77	2	Precuneus_R
45.86	-17.15	10.41	2	Heschl_R
58.15	-21.78	6.8	2	Temporal_Sup_R
48.25	14.75	-16.86	2	Temporal_Pole_Sup_R
-55.52	-33.8	-2.2	2	Temporal_Mid_L
44.22	14.55	-32.23	2	Temporal_Pole_Mid_R
1.15	-64.43	-34.08	2	Vermis_8
41.37	-8.21	52.09	1	Precentral_R
37.59	33.06	34.04	1	Frontal_Mid_R
8.35	35.64	-18.04	1	Rectus_R
27.32	0.64	-17.5	1	Amygdala_R
-7.14	-78.67	6.44	1	Calcarine_L
15.99	-73.15	9.4	1	Calcarine_R
-14.62	-67.56	-4.63	1	Lingual_L
16.29	-66.93	-3.87	1	Lingual_R
-31.16	-40.3	-20.23	1	Fusiform_L
33.97	-39.1	-20.18	1	Fusiform_R
-23.45	-59.56	58.96	1	Parietal_Sup_L
26.11	-59.18	62.06	1	Parietal_Sup_R
-55.79	-33.64	30.45	1	SupraMarginal_L
57.61	-31.5	34.48	1	SupraMarginal_R
-44.14	-60.82	35.59	1	Angular_L
-7.24	-56.07	48.01	1	Precuneus_L

-17.75	-0.03	0.21	1	Pallidum_L
-39.88	15.14	-20.18	1	Temporal_Pole_Sup_L
-36.32	14.59	-34.08	1	Temporal_Pole_Mid_L
-49.77	-28.05	-23.17	1	Temporal_Inf_L
-15	-43.49	-16.93	1	Cerebelum_4_5_L
17.2	-42.86	-18.15	1	Cerebelum_4_5_R
-23.24	-59.1	-22.13	1	Cerebelum_6_L
-22.61	-33.8	-41.76	1	Cerebelum_10_L
1.15	-71.93	-25.14	1	Vermis_7
0.86	-54.87	-34.9	1	Vermis_9
-16.56	47.32	-13.31	0	Frontal_Sup_Orb_L
18.49	48.1	-14.02	0	Frontal_Sup_Orb_R
-30.65	50.43	-9.62	0	Frontal_Mid_Orb_L
33.18	52.59	-10.73	0	Frontal_Mid_Orb_R
-48.43	12.73	19.02	0	Frontal_Inf_Oper_L
50.2	14.98	21.41	0	Frontal_Inf_Oper_R
50.33	30.16	14.17	0	Frontal_Inf_Tri_R
-35.98	30.71	-12.11	0	Frontal_Inf_Orb_L
41.22	32.23	-11.91	0	Frontal_Inf_Orb_R
-47.16	-8.48	13.95	0	Rolandic_Oper_L
52.65	-6.25	14.63	0	Rolandic_Oper_R
-5.32	4.85	61.38	0	Supp_Motor_Area_L
8.62	0.17	61.85	0	Supp_Motor_Area_R
10.43	15.91	-11.26	0	Olfactory_R
-4.8	49.17	30.89	0	Frontal_Sup_Medial_L
39.02	6.25	2.08	0	Insula_R
-4.04	35.4	13.95	0	Cingulum_Ant_L
-4.85	-42.92	24.67	0	Cingulum_Post_L
7.44	-41.81	21.87	0	Cingulum_Post_R
-25.03	-20.74	-10.13	0	Hippocampus_L
-23.27	-0.67	-17.14	0	Amygdala_L
-5.93	-80.13	27.22	0	Cuneus_L
13.51	-79.36	28.23	0	Cuneus_R
-16.54	-84.26	28.17	0	Occipital_Sup_L
24.29	-80.85	30.59	0	Occipital_Sup_R
37.39	-79.7	19.42	0	Occipital_Mid_R
38.16	-81.99	-7.61	0	Occipital_Inf_R
41.43	-25.49	52.55	0	Postcentral_R
-42.8	-45.82	46.74	0	Parietal_Inf_L
46.46	-46.29	49.54	0	Parietal_Inf_R
45.51	-59.98	38.63	0	Angular_R
-7.63	-25.36	70.07	0	Paracentral_Lobule_L
7.48	-31.59	68.09	0	Paracentral_Lobule_R
-23.91	3.86	2.4	0	Putamen_L
27.78	4.91	2.46	0	Putamen_R
21.2	0.18	0.23	0	Pallidum_R
-10.85	-17.56	7.98	0	Thalamus_L
13	-17.55	8.09	0	Thalamus_R
57.47	-37.23	-1.47	0	Temporal_Mid_R
53.69	-31.07	-22.32	0	Temporal_Inf_R
-36.07	-66.72	-28.93	0	Cerebelum_Crus1_L
37.46	-67.14	-29.55	0	Cerebelum_Crus1_R
-28.64	-73.26	-38.2	0	Cerebelum_Crus2_L

32.06	-69.02	-39.95	0	Cerebelum_Crus2_R
-8.8	-37.22	-18.58	0	Cerebelum_3_L
12.32	-34.47	-19.39	0	Cerebelum_3_R
24.69	-58.32	-23.65	0	Cerebelum_6_R
-32.36	-59.82	-45.45	0	Cerebelum_7b_L
33.14	-63.18	-48.46	0	Cerebelum_7b_R
-25.75	-54.52	-47.68	0	Cerebelum_8_L
25.06	-56.34	-49.47	0	Cerebelum_8_R
-10.95	-48.95	-45.9	0	Cerebelum_9_L
9.46	-49.5	-46.33	0	Cerebelum_9_R
25.99	-33.84	-41.35	0	Cerebelum_10_R
0.76	-38.79	-20.05	0	Vermis_1_2
1.38	-39.93	-11.4	0	Vermis_3
1.22	-52.36	-6.11	0	Vermis_4_5
1.14	-67.06	-15.12	0	Vermis_6
0.36	-45.8	-31.68	0	Vermis_10

Supplementary Table S3. Degree of the regions comprising the network that evidenced a positive association with years of formal education using a primary threshhold of $p < 0.01$.

MNI COORDINATES				
X	Y	Z	Degree	AAL Region
-14.62	-67.56	-4.63	32	Lingual_L
-18.45	34.81	42.2	20	Frontal_Sup_L
41.37	-8.21	52.09	19	Precentral_R
7.48	-31.59	68.09	19	Paracentral_Lobule_R
-5.93	-80.13	27.22	18	Cuneus_L
-5.48	-14.92	41.57	17	Cingulum_Mid_L
44.22	14.55	-32.23	16	Temporal_Pole_Mid_R
24.29	-80.85	30.59	14	Occipital_Sup_R
-38.65	-5.68	50.94	12	Precentral_L
21.9	31.12	43.82	12	Frontal_Sup_R
7.44	-41.81	21.87	12	Cingulum_Post_R
13.51	-79.36	28.23	12	Cuneus_R
41.43	-25.49	52.55	12	Postcentral_R
45.86	-17.15	10.41	12	Heschl_R
8.62	0.17	61.85	11	Supp_Motor_Area_R
8.02	-8.83	39.79	11	Cingulum_Mid_R
-7.14	-78.67	6.44	11	Calcarine_L
37.39	-79.7	19.42	11	Occipital_Mid_R
26.11	-59.18	62.06	11	Parietal_Sup_R
16.29	-66.93	-3.87	10	Lingual_R
-36.36	-78.29	-7.84	10	Occipital_Inf_L
-5.08	37.07	-18.14	9	Rectus_L
-16.54	-84.26	28.17	9	Occipital_Sup_L
33.97	-39.1	-20.18	9	Fusiform_R
-23.45	-59.56	58.96	9	Parietal_Sup_L
-4.85	-42.92	24.67	8	Cingulum_Post_L
-7.24	-56.07	48.01	8	Precuneus_L
-36.32	14.59	-34.08	8	Temporal_Pole_Mid_L
-15	-43.49	-16.93	8	Cerebelum_4_5_L
9.1	50.84	30.22	7	Frontal_Sup_Medial_R
-32.39	-80.73	16.11	7	Occipital_Mid_L
48.25	14.75	-16.86	7	Temporal_Pole_Sup_R
-16.56	47.32	-13.31	6	Frontal_Sup_Orb_L
8.35	35.64	-18.04	6	Rectus_R
-7.63	-25.36	70.07	6	Paracentral_Lobule_L
-36.07	-66.72	-28.93	6	Cerebelum_Crus1_L
-47.16	-8.48	13.95	5	Rolandic_Oper_L
-4.8	49.17	30.89	5	Frontal_Sup_Medial_L
-5.17	54.06	-7.4	5	Frontal_Med_Orb_L
-31.16	-40.3	-20.23	5	Fusiform_L
-53.16	-20.68	7.13	5	Temporal_Sup_L
1.22	-52.36	-6.11	5	Vermis_4_5
-33.43	32.73	35.46	4	Frontal_Mid_L
-5.32	4.85	61.38	4	Supp_Motor_Area_L
-23.27	-0.67	-17.14	4	Amygdala_L
15.99	-73.15	9.4	4	Calcarine_R
58.15	-21.78	6.8	4	Temporal_Sup_R

18.49	48.1	-14.02	3	Frontal_Sup_Orb_R
8.16	51.67	-7.13	3	Frontal_Med_Orb_R
-41.99	-18.88	9.98	3	Heschl_L
-39.88	15.14	-20.18	3	Temporal_Pole_Sup_L
-23.24	-59.1	-22.13	3	Cerebelum_6_L
24.69	-58.32	-23.65	3	Cerebelum_6_R
-42.46	-22.63	48.92	2	Postcentral_L
9.98	-56.05	43.77	2	Precuneus_R
57.47	-37.23	-1.47	2	Temporal_Mid_R
-48.43	12.73	19.02	1	Frontal_Inf_Oper_L
-45.58	29.91	13.99	1	Frontal_Inf_Tri_L
-35.98	30.71	-12.11	1	Frontal_Inf_Orb_L
52.65	-6.25	14.63	1	Rolandic_Oper_R
-4.04	35.4	13.95	1	Cingulum_Ant_L
27.32	0.64	-17.5	1	Amygdala_R
38.16	-81.99	-7.61	1	Occipital_Inf_R
-44.14	-60.82	35.59	1	Angular_L
13	-17.55	8.09	1	Thalamus_R
17.2	-42.86	-18.15	1	Cerebelum_4_5_R
-10.95	-48.95	-45.9	1	Cerebelum_9_L
37.59	33.06	34.04	0	Frontal_Mid_R
-30.65	50.43	-9.62	0	Frontal_Mid_Orb_L
33.18	52.59	-10.73	0	Frontal_Mid_Orb_R
50.2	14.98	21.41	0	Frontal_Inf_Oper_R
50.33	30.16	14.17	0	Frontal_Inf_Tri_R
41.22	32.23	-11.91	0	Frontal_Inf_Orb_R
-8.06	15.05	-11.46	0	Olfactory_L
10.43	15.91	-11.26	0	Olfactory_R
-35.13	6.65	3.44	0	Insula_L
39.02	6.25	2.08	0	Insula_R
8.46	37.01	15.84	0	Cingulum_Ant_R
-25.03	-20.74	-10.13	0	Hippocampus_L
29.23	-19.78	-10.33	0	Hippocampus_R
-21.17	-15.95	-20.7	0	ParaHippocampal_L
25.38	-15.15	-20.47	0	ParaHippocampal_R
-42.8	-45.82	46.74	0	Parietal_Inf_L
46.46	-46.29	49.54	0	Parietal_Inf_R
-55.79	-33.64	30.45	0	SupraMarginal_L
57.61	-31.5	34.48	0	SupraMarginal_R
45.51	-59.98	38.63	0	Angular_R
-11.46	11	9.24	0	Caudate_L
14.84	12.07	9.42	0	Caudate_R
-23.91	3.86	2.4	0	Putamen_L
27.78	4.91	2.46	0	Putamen_R
-17.75	-0.03	0.21	0	Pallidum_L
21.2	0.18	0.23	0	Pallidum_R
-10.85	-17.56	7.98	0	Thalamus_L
-55.52	-33.8	-2.2	0	Temporal_Mid_L
-49.77	-28.05	-23.17	0	Temporal_Inf_L
53.69	-31.07	-22.32	0	Temporal_Inf_R
37.46	-67.14	-29.55	0	Cerebelum_Crus1_R
-28.64	-73.26	-38.2	0	Cerebelum_Crus2_L
32.06	-69.02	-39.95	0	Cerebelum_Crus2_R

-8.8	-37.22	-18.58	0	Cerebelum_3_L
12.32	-34.47	-19.39	0	Cerebelum_3_R
-32.36	-59.82	-45.45	0	Cerebelum_7b_L
33.14	-63.18	-48.46	0	Cerebelum_7b_R
-25.75	-54.52	-47.68	0	Cerebelum_8_L
25.06	-56.34	-49.47	0	Cerebelum_8_R
9.46	-49.5	-46.33	0	Cerebelum_9_R
-22.61	-33.8	-41.76	0	Cerebelum_10_L
25.99	-33.84	-41.35	0	Cerebelum_10_R
0.76	-38.79	-20.05	0	Vermis_1_2
1.38	-39.93	-11.4	0	Vermis_3
1.14	-67.06	-15.12	0	Vermis_6
1.15	-71.93	-25.14	0	Vermis_7
1.15	-64.43	-34.08	0	Vermis_8
0.86	-54.87	-34.9	0	Vermis_9
0.36	-45.8	-31.68	0	Vermis_10

Supplementary Table S4. Degree of the regions comprising the network that evidenced a positive association with years of formal education using a primary threshhold of $p < 0.005$.

MNI COORDINATES				
X	Y	Z	Degree	AAL Region
-14.62	-67.56	-4.63	28	Lingual_L
-18.45	34.81	42.2	16	Frontal_Sup_L
41.37	-8.21	52.09	14	Precentral_R
44.22	14.55	-32.23	13	Temporal_Pole_Mid_R
-5.48	-14.92	41.57	12	Cingulum_Mid_L
-5.93	-80.13	27.22	12	Cuneus_L
24.29	-80.85	30.59	11	Occipital_Sup_R
7.48	-31.59	68.09	11	Paracentral_Lobule_R
45.86	-17.15	10.41	10	Heschl_R
7.44	-41.81	21.87	8	Cingulum_Post_R
13.51	-79.36	28.23	8	Cuneus_R
-38.65	-5.68	50.94	7	Precentral_L
8.62	0.17	61.85	7	Supp_Motor_Area_R
-5.08	37.07	-18.14	7	Rectus_L
8.02	-8.83	39.79	7	Cingulum_Mid_R
-4.85	-42.92	24.67	7	Cingulum_Post_L
16.29	-66.93	-3.87	7	Lingual_R
26.11	-59.18	62.06	7	Parietal_Sup_R
21.9	31.12	43.82	6	Frontal_Sup_R
9.1	50.84	30.22	6	Frontal_Sup_Medial_R
-7.14	-78.67	6.44	6	Calcarine_L
-16.54	-84.26	28.17	6	Occipital_Sup_L
37.39	-79.7	19.42	6	Occipital_Mid_R
-36.36	-78.29	-7.84	6	Occipital_Inf_L
33.97	-39.1	-20.18	6	Fusiform_R
41.43	-25.49	52.55	6	Postcentral_R
48.25	14.75	-16.86	6	Temporal_Pole_Sup_R
-36.32	14.59	-34.08	6	Temporal_Pole_Mid_L
-16.56	47.32	-13.31	5	Frontal_Sup_Orb_L
-32.39	-80.73	16.11	5	Occipital_Mid_L
-15	-43.49	-16.93	5	Cerebelum_4_5_L
-23.45	-59.56	58.96	4	Parietal_Sup_L
-7.24	-56.07	48.01	4	Precuneus_L
-36.07	-66.72	-28.93	4	Cerebelum_Crus1_L
1.22	-52.36	-6.11	4	Vermis_4_5
-33.43	32.73	35.46	3	Frontal_Mid_L
-7.63	-25.36	70.07	3	Paracentral_Lobule_L
-23.24	-59.1	-22.13	3	Cerebelum_6_L
18.49	48.1	-14.02	2	Frontal_Sup_Orb_R
-4.8	49.17	30.89	2	Frontal_Sup_Medial_L
8.16	51.67	-7.13	2	Frontal_Med_Orb_R
8.35	35.64	-18.04	2	Rectus_R
-23.27	-0.67	-17.14	2	Amygdala_L
-31.16	-40.3	-20.23	2	Fusiform_L
-41.99	-18.88	9.98	2	Heschl_L
-53.16	-20.68	7.13	2	Temporal_Sup_L
-39.88	15.14	-20.18	2	Temporal_Pole_Sup_L

57.47	-37.23	-1.47	2	Temporal_Mid_R
24.69	-58.32	-23.65	2	Cerebelum_6_R
-48.43	12.73	19.02	1	Frontal_Inf_Oper_L
-45.58	29.91	13.99	1	Frontal_Inf_Tri_L
-35.98	30.71	-12.11	1	Frontal_Inf_Orb_L
-47.16	-8.48	13.95	1	Rolandic_Oper_L
-5.17	54.06	-7.4	1	Frontal_Med_Orb_L
-4.04	35.4	13.95	1	Cingulum_Ant_L
15.99	-73.15	9.4	1	Calcarine_R
13	-17.55	8.09	1	Thalamus_R
58.15	-21.78	6.8	1	Temporal_Sup_R
17.2	-42.86	-18.15	1	Cerebelum_4_5_R
37.59	33.06	34.04	0	Frontal_Mid_R
-30.65	50.43	-9.62	0	Frontal_Mid_Orb_L
33.18	52.59	-10.73	0	Frontal_Mid_Orb_R
50.2	14.98	21.41	0	Frontal_Inf_Oper_R
50.33	30.16	14.17	0	Frontal_Inf_Tri_R
41.22	32.23	-11.91	0	Frontal_Inf_Orb_R
52.65	-6.25	14.63	0	Rolandic_Oper_R
-5.32	4.85	61.38	0	Supp_Motor_Area_L
-8.06	15.05	-11.46	0	Olfactory_L
10.43	15.91	-11.26	0	Olfactory_R
-35.13	6.65	3.44	0	Insula_L
39.02	6.25	2.08	0	Insula_R
8.46	37.01	15.84	0	Cingulum_Ant_R
-25.03	-20.74	-10.13	0	Hippocampus_L
29.23	-19.78	-10.33	0	Hippocampus_R
-21.17	-15.95	-20.7	0	ParaHippocampal_L
25.38	-15.15	-20.47	0	ParaHippocampal_R
27.32	0.64	-17.5	0	Amygdala_R
38.16	-81.99	-7.61	0	Occipital_Inf_R
-42.46	-22.63	48.92	0	Postcentral_L
-42.8	-45.82	46.74	0	Parietal_Inf_L
46.46	-46.29	49.54	0	Parietal_Inf_R
-55.79	-33.64	30.45	0	SupraMarginal_L
57.61	-31.5	34.48	0	SupraMarginal_R
-44.14	-60.82	35.59	0	Angular_L
45.51	-59.98	38.63	0	Angular_R
9.98	-56.05	43.77	0	Precuneus_R
-11.46	11	9.24	0	Caudate_L
14.84	12.07	9.42	0	Caudate_R
-23.91	3.86	2.4	0	Putamen_L
27.78	4.91	2.46	0	Putamen_R
-17.75	-0.03	0.21	0	Pallidum_L
21.2	0.18	0.23	0	Pallidum_R
-10.85	-17.56	7.98	0	Thalamus_L
-55.52	-33.8	-2.2	0	Temporal_Mid_L
-49.77	-28.05	-23.17	0	Temporal_Inf_L
53.69	-31.07	-22.32	0	Temporal_Inf_R
37.46	-67.14	-29.55	0	Cerebelum_Crus1_R
-28.64	-73.26	-38.2	0	Cerebelum_Crus2_L
32.06	-69.02	-39.95	0	Cerebelum_Crus2_R
-8.8	-37.22	-18.58	0	Cerebelum_3_L

12.32	-34.47	-19.39	0	Cerebelum_3_R
-32.36	-59.82	-45.45	0	Cerebelum_7b_L
33.14	-63.18	-48.46	0	Cerebelum_7b_R
-25.75	-54.52	-47.68	0	Cerebelum_8_L
25.06	-56.34	-49.47	0	Cerebelum_8_R
-10.95	-48.95	-45.9	0	Cerebelum_9_L
9.46	-49.5	-46.33	0	Cerebelum_9_R
-22.61	-33.8	-41.76	0	Cerebelum_10_L
25.99	-33.84	-41.35	0	Cerebelum_10_R
0.76	-38.79	-20.05	0	Vermis_1_2
1.38	-39.93	-11.4	0	Vermis_3
1.14	-67.06	-15.12	0	Vermis_6
1.15	-71.93	-25.14	0	Vermis_7
1.15	-64.43	-34.08	0	Vermis_8
0.86	-54.87	-34.9	0	Vermis_9
0.36	-45.8	-31.68	0	Vermis_10

Supplementary Table S5. Degree of the regions comprising the network that evidenced a positive association with years of formal education using a primary threshhold of $p < 0.001$.

MNI COORDINATES				
X	Y	Z	Degree	AAL Region
-14.62	-67.56	-4.63	17	Lingual_L
41.37	-8.21	52.09	6	Precentral_R
-5.93	-80.13	27.22	5	Cuneus_L
-18.45	34.81	42.2	4	Frontal_Sup_L
-5.48	-14.92	41.57	4	Cingulum_Mid_L
44.22	14.55	-32.23	4	Temporal_Pole_Mid_R
21.9	31.12	43.82	3	Frontal_Sup_R
8.62	0.17	61.85	3	Supp_Motor_Area_R
-5.08	37.07	-18.14	3	Rectus_L
-4.85	-42.92	24.67	3	Cingulum_Post_L
7.44	-41.81	21.87	3	Cingulum_Post_R
24.29	-80.85	30.59	3	Occipital_Sup_R
41.43	-25.49	52.55	3	Postcentral_R
45.86	-17.15	10.41	3	Heschl_R
-16.56	47.32	-13.31	2	Frontal_Sup_Orb_L
-7.14	-78.67	6.44	2	Calcarine_L
37.39	-79.7	19.42	2	Occipital_Mid_R
26.11	-59.18	62.06	2	Parietal_Sup_R
-7.24	-56.07	48.01	2	Precuneus_L
7.48	-31.59	68.09	2	Paracentral_Lobule_R
-15	-43.49	-16.93	2	Cerebelum_4_5_L
-38.65	-5.68	50.94	1	Precentral_L
18.49	48.1	-14.02	1	Frontal_Sup_Orb_R
9.1	50.84	30.22	1	Frontal_Sup_Medial_R
8.16	51.67	-7.13	1	Frontal_Med_Orb_R
8.35	35.64	-18.04	1	Rectus_R
8.02	-8.83	39.79	1	Cingulum_Mid_R
16.29	-66.93	-3.87	1	Lingual_R
33.97	-39.1	-20.18	1	Fusiform_R
-23.45	-59.56	58.96	1	Parietal_Sup_L
48.25	14.75	-16.86	1	Temporal_Pole_Sup_R
-23.24	-59.1	-22.13	1	Cerebelum_6_L
1.22	-52.36	-6.11	1	Vermis_4_5
-33.43	32.73	35.46	0	Frontal_Mid_L
37.59	33.06	34.04	0	Frontal_Mid_R
-30.65	50.43	-9.62	0	Frontal_Mid_Orb_L
33.18	52.59	-10.73	0	Frontal_Mid_Orb_R
-48.43	12.73	19.02	0	Frontal_Inf_Oper_L
50.2	14.98	21.41	0	Frontal_Inf_Oper_R
-45.58	29.91	13.99	0	Frontal_Inf_Tri_L
50.33	30.16	14.17	0	Frontal_Inf_Tri_R
-35.98	30.71	-12.11	0	Frontal_Inf_Orb_L
41.22	32.23	-11.91	0	Frontal_Inf_Orb_R
-47.16	-8.48	13.95	0	Rolandic_Oper_L
52.65	-6.25	14.63	0	Rolandic_Oper_R
-5.32	4.85	61.38	0	Supp_Motor_Area_L
-8.06	15.05	-11.46	0	Olfactory_L

10.43	15.91	-11.26	0	Olfactory_R
-4.8	49.17	30.89	0	Frontal_Sup_Medial_L
-5.17	54.06	-7.4	0	Frontal_Med_Orb_L
-35.13	6.65	3.44	0	Insula_L
39.02	6.25	2.08	0	Insula_R
-4.04	35.4	13.95	0	Cingulum_Ant_L
8.46	37.01	15.84	0	Cingulum_Ant_R
-25.03	-20.74	-10.13	0	Hippocampus_L
29.23	-19.78	-10.33	0	Hippocampus_R
-21.17	-15.95	-20.7	0	ParaHippocampal_L
25.38	-15.15	-20.47	0	ParaHippocampal_R
-23.27	-0.67	-17.14	0	Amygdala_L
27.32	0.64	-17.5	0	Amygdala_R
15.99	-73.15	9.4	0	Calcarine_R
13.51	-79.36	28.23	0	Cuneus_R
-16.54	-84.26	28.17	0	Occipital_Sup_L
-32.39	-80.73	16.11	0	Occipital_Mid_L
-36.36	-78.29	-7.84	0	Occipital_Inf_L
38.16	-81.99	-7.61	0	Occipital_Inf_R
-31.16	-40.3	-20.23	0	Fusiform_L
-42.46	-22.63	48.92	0	Postcentral_L
-42.8	-45.82	46.74	0	Parietal_Inf_L
46.46	-46.29	49.54	0	Parietal_Inf_R
-55.79	-33.64	30.45	0	SupraMarginal_L
57.61	-31.5	34.48	0	SupraMarginal_R
-44.14	-60.82	35.59	0	Angular_L
45.51	-59.98	38.63	0	Angular_R
9.98	-56.05	43.77	0	Precuneus_R
-7.63	-25.36	70.07	0	Paracentral_Lobule_L
-11.46	11	9.24	0	Caudate_L
14.84	12.07	9.42	0	Caudate_R
-23.91	3.86	2.4	0	Putamen_L
27.78	4.91	2.46	0	Putamen_R
-17.75	-0.03	0.21	0	Pallidum_L
21.2	0.18	0.23	0	Pallidum_R
-10.85	-17.56	7.98	0	Thalamus_L
13	-17.55	8.09	0	Thalamus_R
-41.99	-18.88	9.98	0	Heschl_L
-53.16	-20.68	7.13	0	Temporal_Sup_L
58.15	-21.78	6.8	0	Temporal_Sup_R
-39.88	15.14	-20.18	0	Temporal_Pole_Sup_L
-55.52	-33.8	-2.2	0	Temporal_Mid_L
57.47	-37.23	-1.47	0	Temporal_Mid_R
-36.32	14.59	-34.08	0	Temporal_Pole_Mid_L
-49.77	-28.05	-23.17	0	Temporal_Inf_L
53.69	-31.07	-22.32	0	Temporal_Inf_R
-36.07	-66.72	-28.93	0	Cerebelum_Crus1_L
37.46	-67.14	-29.55	0	Cerebelum_Crus1_R
-28.64	-73.26	-38.2	0	Cerebelum_Crus2_L
32.06	-69.02	-39.95	0	Cerebelum_Crus2_R
-8.8	-37.22	-18.58	0	Cerebelum_3_L
12.32	-34.47	-19.39	0	Cerebelum_3_R
17.2	-42.86	-18.15	0	Cerebelum_4_5_R

24.69	-58.32	-23.65	0	Cerebelum_6_R
-32.36	-59.82	-45.45	0	Cerebelum_7b_L
33.14	-63.18	-48.46	0	Cerebelum_7b_R
-25.75	-54.52	-47.68	0	Cerebelum_8_L
25.06	-56.34	-49.47	0	Cerebelum_8_R
-10.95	-48.95	-45.9	0	Cerebelum_9_L
9.46	-49.5	-46.33	0	Cerebelum_9_R
-22.61	-33.8	-41.76	0	Cerebelum_10_L
25.99	-33.84	-41.35	0	Cerebelum_10_R
0.76	-38.79	-20.05	0	Vermis_1_2
1.38	-39.93	-11.4	0	Vermis_3
1.14	-67.06	-15.12	0	Vermis_6
1.15	-71.93	-25.14	0	Vermis_7
1.15	-64.43	-34.08	0	Vermis_8
0.86	-54.87	-34.9	0	Vermis_9
0.36	-45.8	-31.68	0	Vermis_10

Supplementary Table S6. Degree of the regions comprising the network that evidenced a negative association with years of formal education using a primary threshhold of p < 0.005.

MNI COORDINATES				
X	Y	Z	Degree	AAL Region
0.36	-45.8	-31.68	36	Vermis_10
0.76	-38.79	-20.05	25	Vermis_1_2
53.69	-31.07	-22.32	4	Temporal_Inf_R
0.86	-54.87	-34.9	4	Vermis_9
-10.95	-48.95	-45.9	3	Cerebelum_9_L
-38.65	-5.68	50.94	2	Precentral_L
41.37	-8.21	52.09	2	Precentral_R
-8.06	15.05	-11.46	2	Olfactory_L
-5.08	37.07	-18.14	2	Rectus_L
8.35	35.64	-18.04	2	Rectus_R
-7.14	-78.67	6.44	2	Calcarine_L
15.99	-73.15	9.4	2	Calcarine_R
-5.93	-80.13	27.22	2	Cuneus_L
13.51	-79.36	28.23	2	Cuneus_R
-14.62	-67.56	-4.63	2	Lingual_L
16.29	-66.93	-3.87	2	Lingual_R
-16.54	-84.26	28.17	2	Occipital_Sup_L
-36.36	-78.29	-7.84	2	Occipital_Inf_L
-31.16	-40.3	-20.23	2	Fusiform_L
33.97	-39.1	-20.18	2	Fusiform_R
41.43	-25.49	52.55	2	Postcentral_R
7.48	-31.59	68.09	2	Paracentral_Lobule_R
-53.16	-20.68	7.13	2	Temporal_Sup_L
44.22	14.55	-32.23	2	Temporal_Pole_Mid_R
17.2	-42.86	-18.15	2	Cerebelum_4_5_R
9.46	-49.5	-46.33	2	Cerebelum_9_R
-16.56	47.32	-13.31	1	Frontal_Sup_Orb_L
18.49	48.1	-14.02	1	Frontal_Sup_Orb_R
52.65	-6.25	14.63	1	Rolandic_Oper_R
8.62	0.17	61.85	1	Supp_Motor_Area_R
10.43	15.91	-11.26	1	Olfactory_R
-5.48	-14.92	41.57	1	Cingulum_Mid_L
8.02	-8.83	39.79	1	Cingulum_Mid_R
24.29	-80.85	30.59	1	Occipital_Sup_R
-32.39	-80.73	16.11	1	Occipital_Mid_L
37.39	-79.7	19.42	1	Occipital_Mid_R
38.16	-81.99	-7.61	1	Occipital_Inf_R
-42.46	-22.63	48.92	1	Postcentral_L
-55.79	-33.64	30.45	1	SupraMarginal_L
-7.63	-25.36	70.07	1	Paracentral_Lobule_L
45.86	-17.15	10.41	1	Heschl_R
58.15	-21.78	6.8	1	Temporal_Sup_R
-55.52	-33.8	-2.2	1	Temporal_Mid_L
57.47	-37.23	-1.47	1	Temporal_Mid_R
-49.77	-28.05	-23.17	1	Temporal_Inf_L
37.46	-67.14	-29.55	1	Cerebelum_Crus1_R
-15	-43.49	-16.93	1	Cerebelum_4_5_L

-22.61	-33.8	-41.76	1	Cerebelum_10_L
-18.45	34.81	42.2	0	Frontal_Sup_L
21.9	31.12	43.82	0	Frontal_Sup_R
-33.43	32.73	35.46	0	Frontal_Mid_L
37.59	33.06	34.04	0	Frontal_Mid_R
-30.65	50.43	-9.62	0	Frontal_Mid_Orb_L
33.18	52.59	-10.73	0	Frontal_Mid_Orb_R
-48.43	12.73	19.02	0	Frontal_Inf_Oper_L
50.2	14.98	21.41	0	Frontal_Inf_Oper_R
-45.58	29.91	13.99	0	Frontal_Inf_Tri_L
50.33	30.16	14.17	0	Frontal_Inf_Tri_R
-35.98	30.71	-12.11	0	Frontal_Inf_Orb_L
41.22	32.23	-11.91	0	Frontal_Inf_Orb_R
-47.16	-8.48	13.95	0	Rolandic_Oper_L
-5.32	4.85	61.38	0	Supp_Motor_Area_L
-4.8	49.17	30.89	0	Frontal_Sup_Medial_L
9.1	50.84	30.22	0	Frontal_Sup_Medial_R
-5.17	54.06	-7.4	0	Frontal_Med_Orb_L
8.16	51.67	-7.13	0	Frontal_Med_Orb_R
-35.13	6.65	3.44	0	Insula_L
39.02	6.25	2.08	0	Insula_R
-4.04	35.4	13.95	0	Cingulum_Ant_L
8.46	37.01	15.84	0	Cingulum_Ant_R
-4.85	-42.92	24.67	0	Cingulum_Post_L
7.44	-41.81	21.87	0	Cingulum_Post_R
-25.03	-20.74	-10.13	0	Hippocampus_L
29.23	-19.78	-10.33	0	Hippocampus_R
-21.17	-15.95	-20.7	0	ParaHippocampal_L
25.38	-15.15	-20.47	0	ParaHippocampal_R
-23.27	-0.67	-17.14	0	Amygdala_L
27.32	0.64	-17.5	0	Amygdala_R
-23.45	-59.56	58.96	0	Parietal_Sup_L
26.11	-59.18	62.06	0	Parietal_Sup_R
-42.8	-45.82	46.74	0	Parietal_Inf_L
46.46	-46.29	49.54	0	Parietal_Inf_R
57.61	-31.5	34.48	0	SupraMarginal_R
-44.14	-60.82	35.59	0	Angular_L
45.51	-59.98	38.63	0	Angular_R
-7.24	-56.07	48.01	0	Precuneus_L
9.98	-56.05	43.77	0	Precuneus_R
-11.46	11	9.24	0	Caudate_L
14.84	12.07	9.42	0	Caudate_R
-23.91	3.86	2.4	0	Putamen_L
27.78	4.91	2.46	0	Putamen_R
-17.75	-0.03	0.21	0	Pallidum_L
21.2	0.18	0.23	0	Pallidum_R
-10.85	-17.56	7.98	0	Thalamus_L
13	-17.55	8.09	0	Thalamus_R
-41.99	-18.88	9.98	0	Heschl_L
-39.88	15.14	-20.18	0	Temporal_Pole_Sup_L
48.25	14.75	-16.86	0	Temporal_Pole_Sup_R
-36.32	14.59	-34.08	0	Temporal_Pole_Mid_L
-36.07	-66.72	-28.93	0	Cerebelum_Crus1_L

-28.64	-73.26	-38.2	0	Cerebelum_Crus2_L
32.06	-69.02	-39.95	0	Cerebelum_Crus2_R
-8.8	-37.22	-18.58	0	Cerebelum_3_L
12.32	-34.47	-19.39	0	Cerebelum_3_R
-23.24	-59.1	-22.13	0	Cerebelum_6_L
24.69	-58.32	-23.65	0	Cerebelum_6_R
-32.36	-59.82	-45.45	0	Cerebelum_7b_L
33.14	-63.18	-48.46	0	Cerebelum_7b_R
-25.75	-54.52	-47.68	0	Cerebelum_8_L
25.06	-56.34	-49.47	0	Cerebelum_8_R
25.99	-33.84	-41.35	0	Cerebelum_10_R
1.38	-39.93	-11.4	0	Vermis_3
1.22	-52.36	-6.11	0	Vermis_4_5
1.14	-67.06	-15.12	0	Vermis_6
1.15	-71.93	-25.14	0	Vermis_7
1.15	-64.43	-34.08	0	Vermis_8

Supplementary Table S7. Degree of the regions comprising the network that evidenced a negative association with years of formal education using a primary threshhold of p < 0.001.

MNI COORDINATES				
X	Y	Z	Degree	AAL Region
0.36	-45.8	-31.68	20	Vermis_10
0.76	-38.79	-20.05	4	Vermis_1_2
-16.54	-84.26	28.17	2	Occipital_Sup_L
-38.65	-5.68	50.94	1	Precentral_L
41.37	-8.21	52.09	1	Precentral_R
52.65	-6.25	14.63	1	Rolandic_Oper_R
8.62	0.17	61.85	1	Supp_Motor_Area_R
-5.08	37.07	-18.14	1	Rectus_L
-5.48	-14.92	41.57	1	Cingulum_Mid_L
8.02	-8.83	39.79	1	Cingulum_Mid_R
-7.14	-78.67	6.44	1	Calcarine_L
-5.93	-80.13	27.22	1	Cuneus_L
13.51	-79.36	28.23	1	Cuneus_R
-14.62	-67.56	-4.63	1	Lingual_L
16.29	-66.93	-3.87	1	Lingual_R
24.29	-80.85	30.59	1	Occipital_Sup_R
-36.36	-78.29	-7.84	1	Occipital_Inf_L
33.97	-39.1	-20.18	1	Fusiform_R
-42.46	-22.63	48.92	1	Postcentral_L
41.43	-25.49	52.55	1	Postcentral_R
-7.63	-25.36	70.07	1	Paracentral_Lobule_L
7.48	-31.59	68.09	1	Paracentral_Lobule_R
-53.16	-20.68	7.13	1	Temporal_Sup_L
44.22	14.55	-32.23	1	Temporal_Pole_Mid_R
-22.61	-33.8	-41.76	1	Cerebelum_10_L
-18.45	34.81	42.2	0	Frontal_Sup_L
21.9	31.12	43.82	0	Frontal_Sup_R
-16.56	47.32	-13.31	0	Frontal_Sup_Orb_L
18.49	48.1	-14.02	0	Frontal_Sup_Orb_R
-33.43	32.73	35.46	0	Frontal_Mid_L
37.59	33.06	34.04	0	Frontal_Mid_R
-30.65	50.43	-9.62	0	Frontal_Mid_Orb_L
33.18	52.59	-10.73	0	Frontal_Mid_Orb_R
-48.43	12.73	19.02	0	Frontal_Inf_Oper_L
50.2	14.98	21.41	0	Frontal_Inf_Oper_R
-45.58	29.91	13.99	0	Frontal_Inf_Tri_L
50.33	30.16	14.17	0	Frontal_Inf_Tri_R
-35.98	30.71	-12.11	0	Frontal_Inf_Orb_L
41.22	32.23	-11.91	0	Frontal_Inf_Orb_R
-47.16	-8.48	13.95	0	Rolandic_Oper_L
-5.32	4.85	61.38	0	Supp_Motor_Area_L
-8.06	15.05	-11.46	0	Olfactory_L
10.43	15.91	-11.26	0	Olfactory_R
-4.8	49.17	30.89	0	Frontal_Sup_Medial_L
9.1	50.84	30.22	0	Frontal_Sup_Medial_R
-5.17	54.06	-7.4	0	Frontal_Med_Orb_L
8.16	51.67	-7.13	0	Frontal_Med_Orb_R

8.35	35.64	-18.04	0	Rectus_R
-35.13	6.65	3.44	0	Insula_L
39.02	6.25	2.08	0	Insula_R
-4.04	35.4	13.95	0	Cingulum_Ant_L
8.46	37.01	15.84	0	Cingulum_Ant_R
-4.85	-42.92	24.67	0	Cingulum_Post_L
7.44	-41.81	21.87	0	Cingulum_Post_R
-25.03	-20.74	-10.13	0	Hippocampus_L
29.23	-19.78	-10.33	0	Hippocampus_R
-21.17	-15.95	-20.7	0	ParaHippocampal_L
25.38	-15.15	-20.47	0	ParaHippocampal_R
-23.27	-0.67	-17.14	0	Amygdala_L
27.32	0.64	-17.5	0	Amygdala_R
15.99	-73.15	9.4	0	Calcarine_R
-32.39	-80.73	16.11	0	Occipital_Mid_L
37.39	-79.7	19.42	0	Occipital_Mid_R
38.16	-81.99	-7.61	0	Occipital_Inf_R
-31.16	-40.3	-20.23	0	Fusiform_L
-23.45	-59.56	58.96	0	Parietal_Sup_L
26.11	-59.18	62.06	0	Parietal_Sup_R
-42.8	-45.82	46.74	0	Parietal_Inf_L
46.46	-46.29	49.54	0	Parietal_Inf_R
-55.79	-33.64	30.45	0	SupraMarginal_L
57.61	-31.5	34.48	0	SupraMarginal_R
-44.14	-60.82	35.59	0	Angular_L
45.51	-59.98	38.63	0	Angular_R
-7.24	-56.07	48.01	0	Precuneus_L
9.98	-56.05	43.77	0	Precuneus_R
-11.46	11	9.24	0	Caudate_L
14.84	12.07	9.42	0	Caudate_R
-23.91	3.86	2.4	0	Putamen_L
27.78	4.91	2.46	0	Putamen_R
-17.75	-0.03	0.21	0	Pallidum_L
21.2	0.18	0.23	0	Pallidum_R
-10.85	-17.56	7.98	0	Thalamus_L
13	-17.55	8.09	0	Thalamus_R
-41.99	-18.88	9.98	0	Heschl_L
45.86	-17.15	10.41	0	Heschl_R
58.15	-21.78	6.8	0	Temporal_Sup_R
-39.88	15.14	-20.18	0	Temporal_Pole_Sup_L
48.25	14.75	-16.86	0	Temporal_Pole_Sup_R
-55.52	-33.8	-2.2	0	Temporal_Mid_L
57.47	-37.23	-1.47	0	Temporal_Mid_R
-36.32	14.59	-34.08	0	Temporal_Pole_Mid_L
-49.77	-28.05	-23.17	0	Temporal_Inf_L
53.69	-31.07	-22.32	0	Temporal_Inf_R
-36.07	-66.72	-28.93	0	Cerebelum_Crus1_L
37.46	-67.14	-29.55	0	Cerebelum_Crus1_R
-28.64	-73.26	-38.2	0	Cerebelum_Crus2_L
32.06	-69.02	-39.95	0	Cerebelum_Crus2_R
-8.8	-37.22	-18.58	0	Cerebelum_3_L
12.32	-34.47	-19.39	0	Cerebelum_3_R
-15	-43.49	-16.93	0	Cerebelum_4_5_L

17.2	-42.86	-18.15	0	Cerebelum_4_5_R
-23.24	-59.1	-22.13	0	Cerebelum_6_L
24.69	-58.32	-23.65	0	Cerebelum_6_R
-32.36	-59.82	-45.45	0	Cerebelum_7b_L
33.14	-63.18	-48.46	0	Cerebelum_7b_R
-25.75	-54.52	-47.68	0	Cerebelum_8_L
25.06	-56.34	-49.47	0	Cerebelum_8_R
-10.95	-48.95	-45.9	0	Cerebelum_9_L
9.46	-49.5	-46.33	0	Cerebelum_9_R
25.99	-33.84	-41.35	0	Cerebelum_10_R
1.38	-39.93	-11.4	0	Vermis_3
1.22	-52.36	-6.11	0	Vermis_4_5
1.14	-67.06	-15.12	0	Vermis_6
1.15	-71.93	-25.14	0	Vermis_7
1.15	-64.43	-34.08	0	Vermis_8
0.86	-54.87	-34.9	0	Vermis_9
